# A case report of solitary fibrous tumor of the thyroid gland and literature review

**DOI:** 10.1097/MD.0000000000034710

**Published:** 2023-08-25

**Authors:** Jin Zhang, Simin Han, Yufeng Zhao, Panpan Song, Hui Zhang, Jiafu Zhang, Detao Ding, Yungang Wu

**Affiliations:** a Clinical Medical College of Jining Medical University, Jining, People’s Republic of China; b Department of Otolaryngology-Head and Neck Surgery, Affiliated Hospital of Jining Medical University, Jining, People’s Republic of China.

**Keywords:** case report, immunohistochemistry, review, solitary fibrous tumor, thyroid gland

## Abstract

**Rationale:**

A solitary fibrous tumor (SFT) is an uncommon soft tissue tumor that was first discovered in the pleura. Although SFTs have been documented in other extra-pleural sites, an SFT in the thyroid gland is highly unusual. An SFT of the thyroid gland can be difficult to diagnose, and there is little information about their Underlying biological behavior.

**Patient concerns:**

We present a case of a 63-year-old man with a progressively growing left-neck mass detected 1 month ago, which was pathologically confirmed to be a benign SFT of the thyroid gland.

**Diagnosis:**

Postoperative pathological examination of the tumor revealed an SFT. Immunopathological examination was consistent with the diagnosis of an SFT.

**Interventions:**

The patient underwent surgical resection of the SFT.

**Outcomes:**

The patient was recurrence-free during 1.5 years of follow-up.

**Lessons:**

Surgical excision is beneficial in SFTs that show no histological signs of malignancy, such as pleomorphism, enhanced mitotic activity, necrosis, bleeding, or capsular invasion. However, because the biologic activity remains unknown, meticulous long-term monitoring is required.

## 1. Introduction

Solitary fibrous tumor (SFT) is an uncommon mesenchymal spindle cell neoplasm. Klemperer and Rabin initially described 5 SFTs of the pleura in 1931,^[1]^ and since then, its occurrence in several extra-pleural areas has been reported, including the meninges, eyelids, orbit, paranasal sinuses, and nose. SFTs in the thyroid gland are particularly uncommon, with only 42 occurrences reported in the literature. Taccagni et al^[2]^ reported the first thyroid SFT in 1993. Although 13% to 15% of pleural SFTs are malignant, extra-pleural SFTs are typically benign.^[3]^ There is little known about the ultrasound findings of an SFT of the thyroid gland. Herein, we reviewed the clinical data of a patient with SFT in the thyroid gland and reviewed the relevant literature to improve the understanding of this disease and provide clinical evidence for further diagnosis and treatment of this disease.

## 2. Illustrative case report

**Figure 1.**
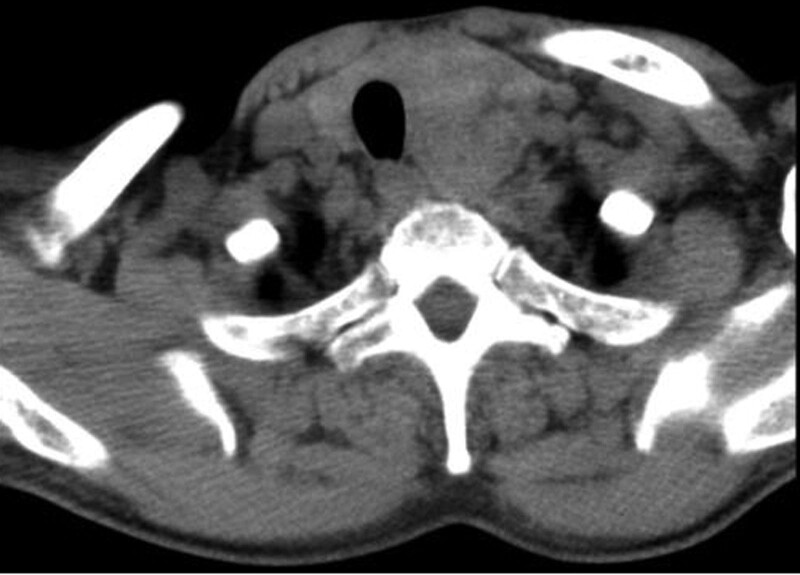
A heterogeneous echoic mass was seen in the left lobe of the thyroid gland.

**Figure 2.**
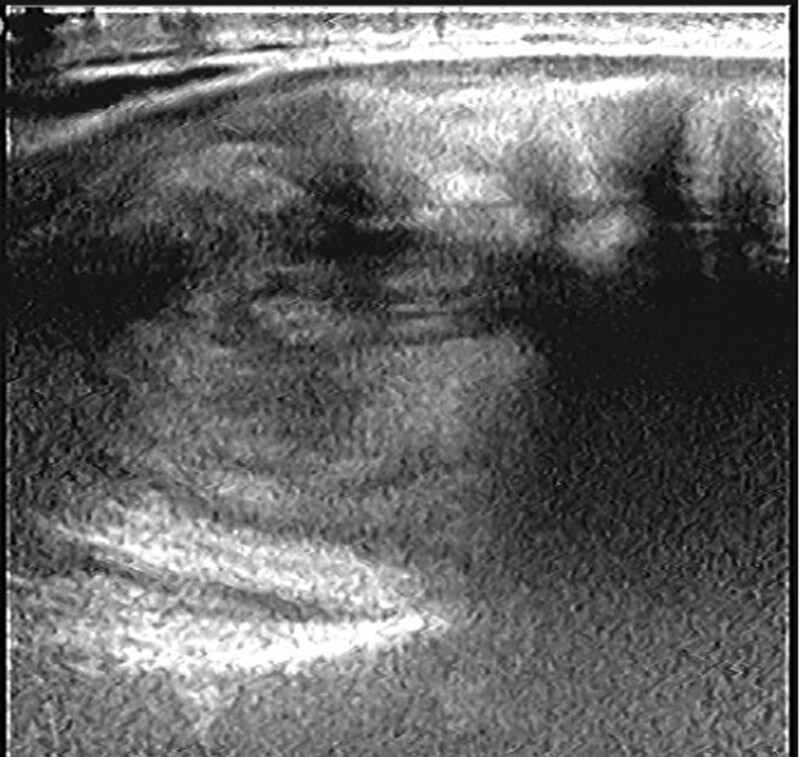
Abundant blood flow signal can be detected in the tumor.

**Figure 3.**
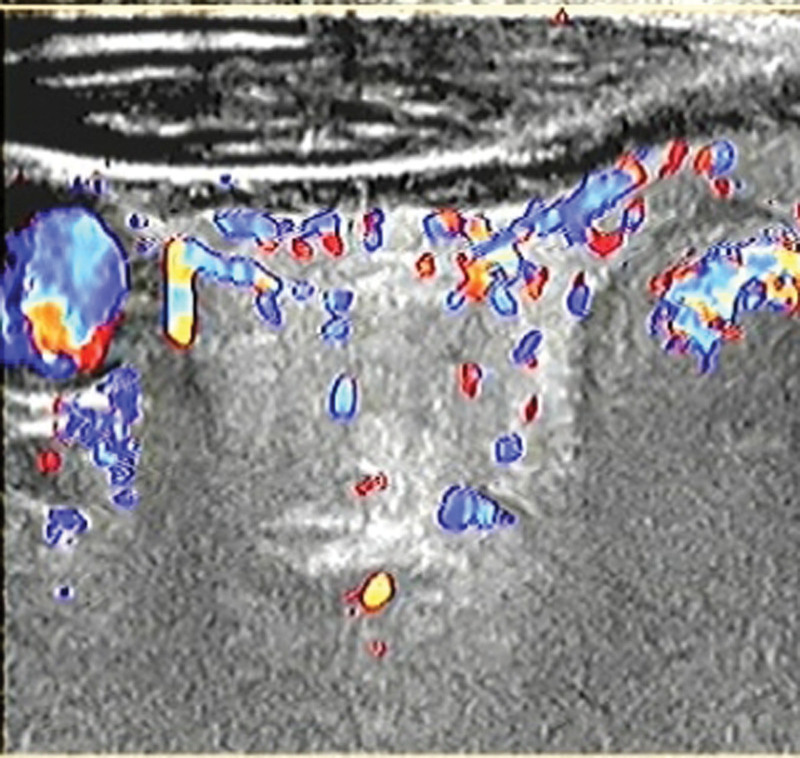
The left lobe of the thyroid gland showing a slightly low-density shadow, uneven density, heterogeneous enhancement. Some small flaky areas without enhancement were noted.

A 63-year-old man was admitted to the hospital for a solid nodule in his left thyroid lobe that was discovered during a physical examination a month ago. He had no significant past medical history. Because the mass did not cause symptoms of dyspnea, dysphonia, or dysphagia, it was not an evident or noticeable concern to the patient. The patient showed no weight loss, pain, or changes in overlying skin and had no related family history of thyroid disease. His neck was asymmetrical, and the trachea was right-sided. The physical exam revealed a 5 cm × 2 cm mass on the left side of the left neck. It was tough and non-tender, had well-defined boundaries, and moved up and down with swallowing. There was no evident mass on the right lobe and no enlarged lymph nodes in the neck. The findings of thyroid function tests were normal. Thyroid-stimulating hormone, free triiodothyronine, free thyroxine, and thyroperoxidase antibodies were within normal limits. Computed tomography (CT) of the neck revealed a heterogeneously enhanced mass with uneven density arising from the thyroid gland’s left lobe. The mass measured 6.0 cm × 3.9 cm × 3.4 cm and compressed the surrounding tissues and shifted the trachea to the right. No significant abnormalities were noted in the right lobe of the thyroid gland. Contrary to the physical examination, CT revealed several slightly enlarged lymph nodes in the submental, submandibular, and bilateral carotid sheath areas. The CT scan and enhancement scan of the thyroid gland revealed a slightly hypointense shadow in the left lobe of the thyroid gland and heterogeneous delayed enhancement with a “fast-in, slow-out” feature (Fig. 1). Color Doppler ultrasound revealed a solid heterogeneous echogenic mass measuring 5.8 cm × 4.0 cm × 2.7 cm in the left lobe of the thyroid gland (Fig. 2). The mass had clear borders, regular morphology, heterogeneous internal echogenicity, posterior echogenic attenuation, and an abundant branching blood flow signal (Fig. 3). Pulsed-wave Doppler US is an arterial-like spectrum. Thyroid Imaging Reporting and Data System grades level IV.

**Figure 4.**
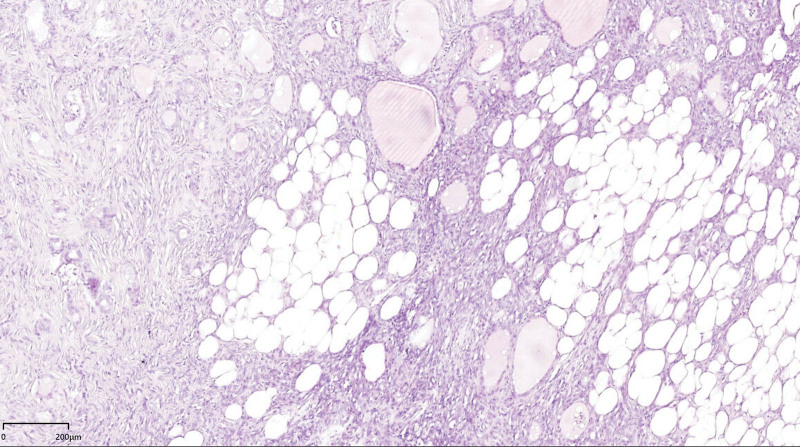
Spindle cells forming a “patternless” growth architecture intermingled with hypercellular and hypocellular areas, and abundant presence of adipocytes (fat-forming variant) was noted. (HE × 4).

**Figure 5.**
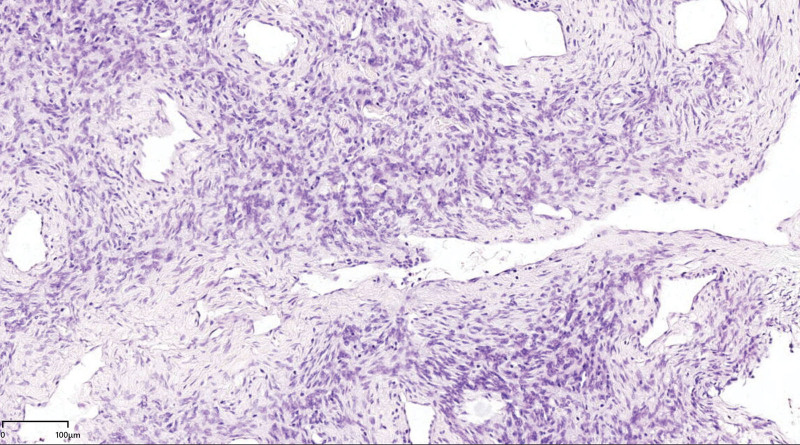
Tumor cells showed no obvious nuclear atypia and no clear nuclear division; numerous branched parenchyma blood vessels were visible, and some perivascular inflammatory cells were clustered. (HE × 20).

**Figure 6.**
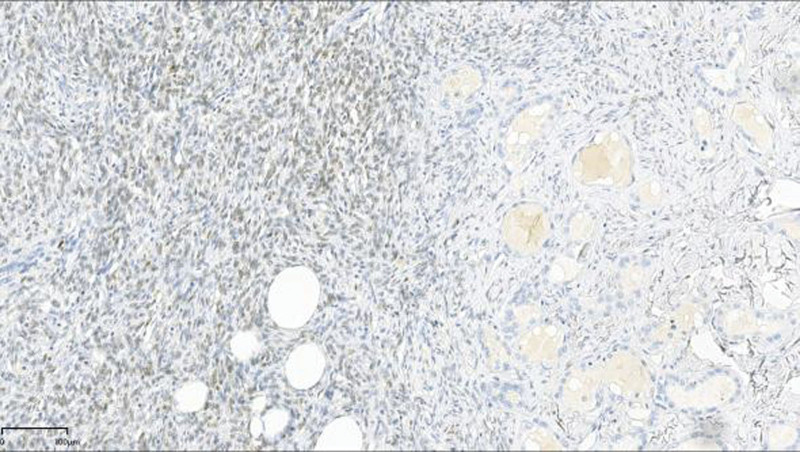
Immunohistochemical analysis of STAT6 showed diffuse nuclear expression in tumor cells. (EnVision, ×10).

**Figure 7.**
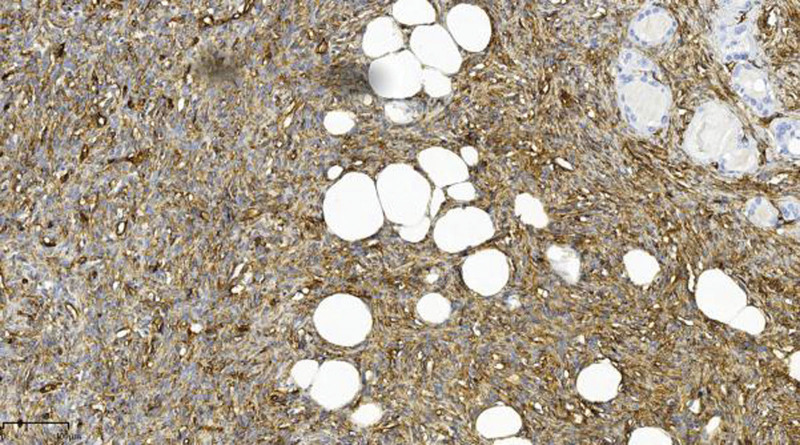
Immunohistochemical staining showed strong positive expression of CD34 in tumor cells. (EnVision, ×10).

Thereafter, the patient underwent a subtotal thyroid lobectomy without adjuvant treatment. The left lobe of the thyroid gland was removed, which was then identified to have a mass with a tough texture, a smooth surface, and clear boundaries; the right lobe, however, had no mass. Histological analysis of intraoperatively frozen sections of the mass revealed spindle cell tumor with abundant cells, occasional mitosis, and no definite necrosis. During the operation, the left laryngeal nerve and upper and lower parathyroid glands were carefully protected. The ultimate diagnosis on the basis of pathologic and immunohistochemical findings was of an SFT of the thyroid gland. On gross examination, the resected tumor measured 6.0 cm × 4.2 cm × 3.0 cm, and it was well-defined, limited to the thyroid gland, and off-white in color. Histologically, the tumor was highly cellular, with spindle cells generating a “patternless” growth architecture but with a collagenous stroma and a thin-walled, branched “staghorn” vessels’ configuration. Adipocytotic (fat-forming variant) and normal thyroid follicles were noted to be in abundance (Fig. 4). There was no evidence of necrosis, cellular pleomorphism, or clear mitotic figures (Fig. 5). Immunohistochemistry revealed diffuse nuclear STAT6 expression (Fig. 6), positive CD34 (Fig. 7) and CD99 expression, and negative S-100, desmin, and CD117 expression. The proliferation index Ki-67 was positive in less than 1% of the tumor cells. A pathological diagnosis of a lipogenic isolated fibrous tumor of the left lobe of the thyroid was made. The patient was discharged from the hospital on the fifth day after surgery and was discharged without any antibiotics, but was instructed to take one oral levothyroxine sodium tablet daily for 6 months. He had been followed up for 1.5 years since the operation, and ultrasound and CT have revealed no signs of recurrence or metastasis.

## 3. Literature review

We evaluated the available literature on SFTs of the thyroid gland. Several PubMed searches were performed to identify patients with this condition in the published literature. The search terms used were *solitary fibrous tumor, thyroid gland, head and neck carcinoma, intrathoracic goiter*, and *thyroid gland mass*. Additional cases were discovered by checking the references cited by the articles obtained using our search. The search period was set to 1993 to present, and we limited our search to research published in English. Taken together, we found 42 reports of an SFT of the thyroid gland. In this review of literature, we examined the clinical signs, imaging characteristics, differential diagnosis, histology, immunohistochemistry, treatment, and prognosis (Table 1).^[4–8]^

**Table 1:** Summary of the clinicopathological features in the reported cases of solitary fibrous tumor derived from the thyroid gland.

Case	Year	Ref.	Age	Sex	Site	Size (cm)	Atypia	Mitosis (>4/10HPF)	Necrosis	STAT6	CD34	Treatment	F/U (mo)
1	1993	Taccagni G et al	44	F	R	6	No	Rare	NA	NA	NA	Lobectomy	NED (60)
2	1993	Taccagni G et al	61	M	L	6	No	No	NA	NA	NA	Subtotal	NED (48)
3	1993	Taccagni G et al	32	F	R	3.5	Yes	Rare	NA	NA	NA	Lobectomy	NED (60)
4	1997	Kie JH et al	48	F	R	8	No	No	NA	NA	Yes	Subtotal	NA
5	1999	Brunnemann RB et al	28	F	NA	2.5	NA	No	NA	NA	NA	NA	NA
6	2001	Rodriguez et al	43	F	L	3.5	No	No	No	NA	Yes	NA	NA
7	2001	Rodriguez et al	52	M	L	2.5	No	No	No	NA	Yes	NA	NA
8	2001	Rodriguez et al	44	M	L	2	Yes/mild	No	No	NA	Yes	NA	NA
9	2001	Rodriguez et al	64	F	R	4.5	No	No	No	NA	Yes	NA	NED (60)
10	2001	Rodriguez et al	53	M	L	6	No	No	No	NA	Yes	NA	NED (60)
11	2001	Rodriguez et al	47	F	R	4.5	No	No	No	NA	Yes	NA	NED (48)
12	2001	Rodriguez et al	64	F	L	3	No	No	No	NA	Yes	NA	NA
13	2001	Deshmukh NS et al	56	M	R	8	No	No	No	NA	Yes	hemithyroidectomy	NED (12)
14	2003	Bohórquez CL et al	68	M	L	9.7	No	No	No	NA	Yes	Subtotal	NED (54)
15	2003	Parwani AV et al	61	M	L	5	N	NA	No	NA	Yes	Subtotal	NA
16	2004	Babouk NL.	45	M	L	5	No	No	No	NA	Yes	hemithyroidectomy	NA
17	2006	Tanahashi J et al	64	M	R	5	No	No	No	NA	NA	Subtotal	NED (60)
18	2006	Tanahashi J et al	41	M	R	3	No	No	No	NA	Yes	hemithyroidectomy	NED (48)
19	2006	Papi G et al	70	F	R	1.7	No	No	No	NA	Yes	Total	NED (6)
20	2007	Papi et al	45	M	L	5	No	No	No	NA	Yes	Total	NED (6)
21	2008	Santeusanio G et al	61	M	R	3.5	NA	No	No	NA	Yes	total	NED (60)
22	2008	Santeusanio G et al	42	F	R	4.7	NA	No	No	NA	Yes	hemithyroidectomy with isthmusectomy	NED (84)
23	2009	Farrag et al	51	M	L	7	NA	NA	NA	NA	Yes	Lobectomy	NA
24	2010	Cameselle-Teijeiro J.	43	M	L	4	No	No	No	NA	Yes	Subtotal	NED(252)
25	2010	Ning et al	76	F	R	4	Yes	High	Yes	NA	Yes	Lobectomy	RC/ MT (5)
26	2010	Larsen SR et al	58	M	IT	8	No	Low	No	NA	Yes	expanded hemi-thyroidectomy	NA
27	2010	Cox DP et al	69	F	NA	2.2	NA	NA	NA	NA	Yes	NA	NA
28	2011	Song Z et al	37	M	L	4	No	No	No	NA	Yes	Subtotal	NED (16)
29	2011	Verdi D et al	47	F	L	5.2	NA	NA	No	NA	Yes	Lobectomy	NED (9)
30	2011	Verdi D et al	59	M	R	7	No	No	No	NA	Yes	Lobectomy	NED (31)
31	2013	Lin MW et al	88	F	IT	9	NA	No	Yes	NA	Yes	Subtotal	NED (36)
32	2013	Oya Topaloglu	68	M	L	8.5	NA	NA	NA	NA	Yes	tumorectomy	NED(9)
33	2013	Alves Filho et al	60	F	R	13.8	NA	Yes	No	NA	No	hemithyroidectomy	NA
34	2014	Mohammad Vaziri et al	74	M	IT	12	No	No	No	NA	Yes	near total	NED(24)
35	2014	Mizuuchi Y et al	78	M	R	3.5	No	No	No	NA	Yes	Subtotal	NED (12)
36	2014	Boonlorm N	61	F	L	10.5	No	No	No	NA	NA	Subtotal	NED (19)
37	2019	Ghasemi-Rad M et al	41	F	L	11	moderate	High	No	Yes	Yes	Total	NED (10)
38	2019	Thompson et al	44	F	NA	7	No	1/3	No	Yes	2/3	Lobectomy	NED (41)
39	2019	Thompson et al	45	F	NA	8.2	No	1例	No	Yes	2例	Lobectomy	NED (28)
40	2019	Thompson et al	52	M	NA	7	No		No	Yes		Lobectomy	NED (5)
41	2019	Suh YJ et al	59	M	L	5.5	Yes	Yes	No	Yes	Yes	Lobectomy	NED (17)
42	2021	Negură I	34	M	L	5.1	NA	No	No	NA	Yes	Total	NA
43	2022	Prensent	68	M	L	6.2	No	No	No	Yes	Yes	Subtotal	NED (18)

F = female, F/U = follow up, IT = intrathoracic, L = LEFT lobe, M = male, NA = not available, NED = no evidence of disease, R = right lobe, RC/MT.

The age range of the reported patients (19 women and 24 men) was 28 to 88 years (mean age: 54.5 years), and the diameter of the neoplasm ranged from 23 to 138 mm (mean diameter: 58 mm). The SFT was located in the right lobe in 15 cases and the left lobe in 20 cases; however, its location was not reported in 5 cases. Three cases were detected to have retrosternal thyroid occupancy. SFT has no distinct clinical signs and is most commonly described as a painless, slow-growing mass that may cause symptoms resulting from compression of adjacent tissues, such as dysphagia, dyspnea, and dysphonia. The tumor may occasionally migrate downhill from the thyroid gland and present as a retrosternal thyroid occupancy. In the published cases reviewed by us, patients with SFT of the thyroid gland underwent hemithyroidectomy, partial thyroidectomy, or complete thyroidectomy without chemotherapy. To our knowledge, the SFT of the thyroid gland appeared as a well-circumscribed and solid lesion in most cases, with most patients not presenting with hemorrhage, necrosis, atypia, mitosis, or calcification. The STAT6 immunohistochemistry results were unknown in 37 patients and positive in 6 patients. Among the 43 patients, CD 34 positivity was present in 35 patients, absent in 2 patients, and unknown in 6 patients. To our knowledge, there have only been 2 reports of malignant SFT in the thyroid gland, one of which reported local recurrence and bilateral lung metastases 5 months postoperatively.^[9]^ In the other case, there were no intraoperative symptoms of malignancy, and the patient had preoperative hoarseness and no postoperative follow-up information.^[10]^ We reviewed the medical records of 42 SFTs of the thyroid gland published between 1993 and 2022. Among them, follow-up information was missing for 13 patients. Of these, 28 patients were followed up for a mean duration of 40 months (range, 5–252 months), and within their respective follow-up periods, none of these patients showed recurrence or metastasis.

## 4. Discussion

Although SFT cannot always be confirmed by imaging, it cannot be ignored. It is a mass with iso- or hypo-density relative to the musculature and brain on T1-weighted MR images, and it is homogenously hyperintense or occasionally heterogeneous depending on cellularity, collagen quantity, and the presence of degeneration or hemorrhage on T2-weighted images.^[11]^ CT scan is widely used to evaluate pleural and extrapleural SFT to examine tumor expansion into surrounding tissues and surgical planes. The tumor has different densities on the CT scan depending on the amount of collagen and myxoid tissue present.^[12]^ On enhanced images, it frequently exhibits rich vascularization of the neoplasm.^[13]^ The diagnosis of an SFT of the thyroid gland is challenging and may not be accurately indicated by its clinical manifestations or imaging test findings. An SFT of the thyroid gland can be difficult to distinguish from other tumors, such as anaplastic carcinomas, hemangiopericytomas, medullary carcinomas, sarcomas, leiomyomas, neurofibromas, schwannomas, and Riedel’s thyroiditis.^[7,10,14,15]^ A diagnosis of an SFT of the thyroid gland can only be made after the exclusion of other thyroid tumors that exhibit spindle-cell morphology.^[16]^

SFTs of the thyroid gland have a distinct morphology that is often referred to as a “patternless” pattern, showing spindle cell growth interspersed with hypercellular and hypocellular patches.^[10]^ Present within areas of the tumor were filled thyroid gland follicles of different sizes. SFTs are histologically characterized by proliferative ovoid to spindle-shaped tumor cells surrounded by intermittent staghorn vessels and structured with a fibrocollagenous to myxoid matrix. Factors like the proportion of cellular versus stromal components, the presence of adipocytic (fat-forming variant) and multinucleated cells which results in a giant-cell angiofibroma-like appearance, and variations in nuclear atypia and mitosis all contribute to a wide presentation range of SFTs in terms of histomorphology and tumor behavior.

The diagnosis of SFTs requires combined assessment of CD34 and STAT6 in a proper clinicopathological context.^[17]^ Indeed, the stated sensitivity of STAT6 nuclear expression in diagnosing SFTs was not always 100%. The function of CD34 and Bcl-2 expression in SFT cells remains unknown. The former suggests that these cells are derived from bone marrow precursor cells, whereas the latter suggests that reduced apoptosis may result in increased cell quantity, eventually leading to cancer.^[4]^ STAT6 immunohistochemistry reportedly has extremely high sensitivity and specificity for the presence of the NAB2–STAT6 gene fusion product, making STAT6 an excellent immunohistochemical marker for validating the diagnosis of an SFT.^[18]^ Thus, we used immunohistochemistry to get a definitive diagnosis.

Surgical excision is currently the primary treatment for SFTs, and operation is performed in most patients to acquire a clear diagnosis and relieve tracheal and/or esophageal compression. If no malignancy is identified, the scope of the surgery should be reduced to minimize the risk of harm to perithyroidal tissues.^[4]^ This is a particularly significant approach for the thyroid region wherein more aggressive surgery can cause significant morbidity. Alves Filho et al recommended that in situations of preoperative vocal fold impairment, the surgical process should begin with exploring the paresis side.

In terms of the prognosis, local recurrence or distant metastases local recurrence or distant metastases occur in 10% to 15% of patients with intrathoracic SFT. High cellularity, moderate-to-strong cytological atypia, a higher frequency of mitoses (>4/10 HPF), and signs of tumor necrosis or infiltrating margins were some of the criteria first proposed by Vallat-Decouvelaere et al in 1998.^[19]^ Because these tumors are uncommon, particularly in cases of malignancy, little is known regarding their behavior or prognosis. Close monitoring after surgical excision of lesions appears to be the most effective management.

## 5. Conclusion

In conclusion, we herein described a case of thyroid SFT. Although uncommon, if a slowly increasing thyroid tumor is identified, SFT should be considered during differential diagnosis. The current patient showed no histological signs of malignancy, such as pleomorphism, enhanced mitotic activity, necrosis, bleeding, or capsular invasion, and at 1.5 years postoperatively, no recurrence or metastasis was noted. However, because the biologic activity is yet unknown, meticulous long-term monitoring is required.

## Author contributions

**Investigation:** Panpan Song, Jiafu Zhang.

**Resources:** Yungang Wu.

**Supervision:** Yufeng Zhao, Panpan Song, Jiafu Zhang.

**Writing – original draft:** Jin Zhang, Simin Han.

**Writing – review & editing:** Yufeng Zhao, Hui Zhang, Detao Ding, Yungang Wu.
